# Acridine 0.75-hydrate

**DOI:** 10.1107/S1600536811038220

**Published:** 2011-09-30

**Authors:** Einat Schur, Joel Bernstein, Andreas Lemmerer, Radion Vainer

**Affiliations:** a Ben Gurion University of the Negev, Beer Sheva, Israel 84105

## Abstract

The title compound, C_13_H_9_N·0.75H_2_O was obtained during a study of the polymorphic system of acridine, by slow evaporation from an ethanol–water solution. There are two acridine mol­ecules (indicated by I and II, respectively) and one and a half water mol­ecules in the asymmetric unit. The half-mol­ecule of water is located on a crystallographic twofold axis. The crystal structure is built up from two threads of mol­ecule II sewn together with water mol­ecules through O—H⋯O and O—H⋯N hydrogen bonds from one side and with π–π inter­actions [centroid–centroid distance = 3.640 (3) and 3.7431 (3) Å] between overlapping mol­ecules II on the other side. Mol­ecule I is attached to this thread from both sides by C—H⋯O hydrogen bonds. The threads are connected to each other by π–π inter­actions [centroid–centroid distances = 3.582 (3) and 3.582 (3) Å] between the inner side of mol­ecule I and stabilized by a C—H⋯π inter­action on the other side of mol­ecule I. This thread with rows of mol­ecule I hanging on its sides is generated by translation perpendicular to the *a* axis.

## Related literature

For the five anhydrous polymorphs of acridine, see: Phillips (1954 ▶, 1956 ▶), Phillips *et al.* (1960 ▶) and Mei & Wolf (2004 ▶) for monoclinic forms VI and VII, and Braga *et al.* (2010 ▶) for ortho­rhom­bic form IV and monoclinic forms II and III. For further crystallographic studies of acridine hydrate, see: Groth (1919 ▶); Lowde *et al.* (1953 ▶). 
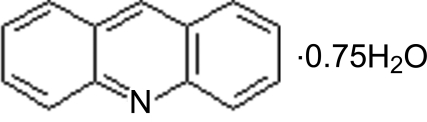


## Experimental

### 

#### Crystal data

C_13_H_9_N·0.75H_2_O
*M* *_r_* = 192.71
Orthorhombic, 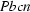

*a* = 26.400 (5) Å
*b* = 8.893 (5) Å
*c* = 17.492 (5) Å
*V* = 4107 (3) Å^3^
*Z* = 16
Mo *K*α radiation
μ = 0.08 mm^−1^
*T* = 197 K
0.3 × 0.3 × 0.3 mm

#### Data collection

Bruker SMART 6000 diffractometer
14504 measured reflections
3606 independent reflections
1733 reflections with *I* > 2σ(*I*)
*R*_int_ = 0.068

#### Refinement

*R*[*F* ^2^ > 2σ(*F* ^2^)] = 0.058
*wR*(*F* ^2^) = 0.197
*S* = 1.00
3606 reflections
272 parameters
2 restraints
H atoms treated by a mixture of independent and constrained refinement
Δρ_max_ = 0.35 e Å^−3^
Δρ_min_ = −0.29 e Å^−3^

### 

Data collection: *SMART* (Bruker, 2005 ▶); cell refinement: *SAINT* (Bruker, 2003 ▶); data reduction: *SAINT*; program(s) used to solve structure: *SHELXTL* (Sheldrick, 2008 ▶); program(s) used to refine structure: *SHELXTL*; molecular graphics: *OLEX2* (Dolomanov *et al.*, 2009 ▶); software used to prepare material for publication: *OLEX2*.

## Supplementary Material

Crystal structure: contains datablock(s) I, global. DOI: 10.1107/S1600536811038220/ez2255sup1.cif

Structure factors: contains datablock(s) I. DOI: 10.1107/S1600536811038220/ez2255Isup2.hkl

Supplementary material file. DOI: 10.1107/S1600536811038220/ez2255Isup3.cml

Additional supplementary materials:  crystallographic information; 3D view; checkCIF report

## Figures and Tables

**Table 1:** Hydrogen-bond geometry (Å, °) *Cg*1 and *Cg*2 are the centroids of the C1/C6–C8/C13/N1 and C1–C6 rings, respectively.

*D*—H⋯*A*	*D*—H	H⋯*A*	*D*⋯*A*	*D*—H⋯*A*
O2—H2*B*⋯N2	0.933 (3)	1.942 (2)	2.873 (4)	175.2 (2)
C7—H7⋯O1	0.93	2.35	3.271 (4)	171
O1—H1⋯O2^i^	0.95 (1)	1.98 (5)	2.777 (4)	139 (6)
C16—H16⋯*Cg*1^ii^	0.93	2.93	3.773 (5)	152
C18—H18⋯*Cg*2^iii^	0.93	2.93	3.848 (5)	168

Symmetry codes: (i) 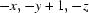; (ii) 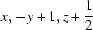; (iii) 
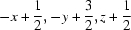
.

**Fig. 1.**
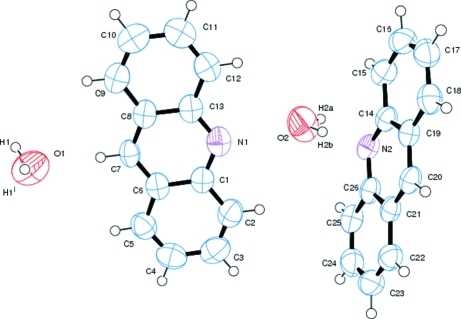
The asymmetric unit with atom labels and 50% probability displacement ellipsoids for non-H atoms. Atom H1i is generated by a two-fold axis (-x, y, -z-1/2).

**Fig. 2.**
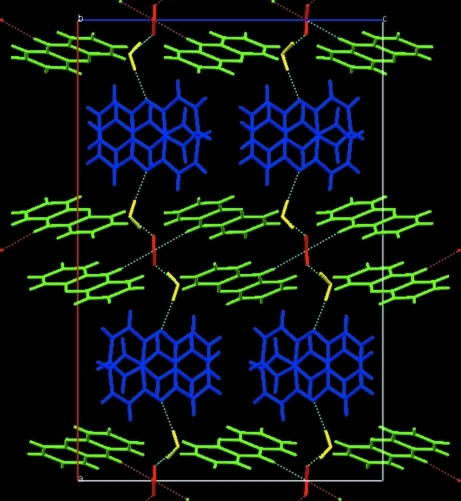
The packing of acridine hydrate viewed down the b axis. Hydrogen bonds are marked in dashed lines.

## supplementary crystallographic information

### Comment

Acridine hydrate is the hydrated form of the very rich polymorphic system of acridine. There are five anhydrous polymorphs of acridine with fully analyzed structures: an orthorhombic form and four monoclinic forms. For the orthorhombic form (form IV) cell parameters were first published by Phillips (1954), and the full solution was recently published by Braga *et al.* (2010). The monoclinic forms are designated II, III, VI and VII. The crystal structure of forms III and II respectively were determined by Phillips (1956) and Phillips *et al.* (1960) and redetermined by Mei and Wolf (2004) and by Braga *et al.* (2010). Forms VI and VII were reported by Mei and Wolf (2004). The form described in this paper was initially thought to be one of the first polymorphs of acridine and known historically as the orthorhombic form of Groth (1919) and subsequently labeled as acridine I. Lowde *et al.* (1953) established the unit cell parameters, the space group and the density. From analysis using the Karl Fischer reagent, it was concluded that acridine I is in fact the monohydrate and not a polymorph of acridine.

There are two acridine molecules and one and a half water molecules in the asymmetric unit (see Fig. 1). In the packing diagram (see Fig. 2), molecule I is colored in green, molecule II is colored in blue, the water molecule that is sitting on a two fold axis is red and the other one is in yellow. The molecules are linked by O—H···O and C—H···O hydrogen bonds (see Table 1).

### Experimental

The title compound was obtained by slow evaporation from an ethanol-water solution in 3:1 and 2:1 ratio at 4°C. The crystals are unstable at room temperature and transform to the anhydrous form III. The common habit of acridine hydrate is thick yellow needles but other habits may be obtained as well.

### Refinement

The water H atoms were located in a difference map and refined with distance restraints of O—H = 0.94 (2) Å. Other H atoms were positioned geometrically and refined using a riding model with C—H = 0.930 (1) Å.

### Crystal data

**Table:**

C_13_H_9_N·0.75H_2_O	*F*(000) = 1632
*M**_r_* = 192.71	*D*_x_ = 1.247 Mg m^−^^3^*D*_m_ = 1.247 Mg m^−^^3^*D*_m_ measured by not measured
Orthorhombic, *P**b**c**n*	Mo *K*α radiation, λ = 0.71073 Å
Hall symbol: -P 2n 2ab	Cell parameters from 1764 reflections
*a* = 26.400 (5) Å	θ = 2.3–21.8°
*b* = 8.893 (5) Å	µ = 0.08 mm^−^^1^
*c* = 17.492 (5) Å	*T* = 197 K
*V* = 4107 (3) Å^3^	Cube, yellow
*Z* = 16	0.3 × 0.3 × 0.3 mm

### Data collection

**Table:**

Bruker SMART 6000 diffractometer	1733 reflections with *I* > 2σ(*I*)
Radiation source: fine-focus sealed tube	*R*_int_ = 0.068
graphite	θ_max_ = 25.0°, θ_min_ = 3.3°
phi and ω scans	*h* = −31→22
14504 measured reflections	*k* = −10→9
3606 independent reflections	*l* = −20→20

### Refinement

**Table:**

Refinement on *F*^2^	Primary atom site location: structure-invariant direct methods
Least-squares matrix: full	Secondary atom site location: difference Fourier map
*R*[*F*^2^ > 2σ(*F*^2^)] = 0.058	Hydrogen site location: inferred from neighbouring sites
*wR*(*F*^2^) = 0.197	H atoms treated by a mixture of independent and constrained refinement
*S* = 1.00	*w* = 1/[σ^2^(*F*_o_^2^) + (0.0837*P*)^2^ + 1.779*P*] where *P* = (*F*_o_^2^ + 2*F*_c_^2^)/3
3606 reflections	(Δ/σ)_max_ = 0.035
272 parameters	Δρ_max_ = 0.35 e Å^−^^3^
2 restraints	Δρ_min_ = −0.29 e Å^−^^3^

### Special details

**Table:**

Geometry. All e.s.d.'s (except the e.s.d. in the dihedral angle between two l.s. planes) are estimated using the full covariance matrix. The cell e.s.d.'s are taken into account individually in the estimation of e.s.d.'s in distances, angles and torsion angles; correlations between e.s.d.'s in cell parameters are only used when they are defined by crystal symmetry. An approximate (isotropic) treatment of cell e.s.d.'s is used for estimating e.s.d.'s involving l.s. planes.
Refinement. Refinement of *F*^2^ against ALL reflections. The weighted *R*-factor *wR* and goodness of fit *S* are based on *F*^2^, conventional *R*-factors *R* are based on *F*, with *F* set to zero for negative *F*^2^. The threshold expression of *F*^2^ > σ(*F*^2^) is used only for calculating *R*-factors(gt) *etc*. and is not relevant to the choice of reflections for refinement. *R*-factors based on *F*^2^ are statistically about twice as large as those based on *F*, and *R*- factors based on ALL data will be even larger.

### Fractional atomic coordinates and isotropic or equivalent isotropic displacement parameters (Å^2^)

**Table:**

	*x*	*y*	*z*	*U*_iso_*/*U*_eq_	
C1	0.08797 (12)	0.6902 (4)	−0.0316 (2)	0.0579 (9)	
C2	0.10417 (14)	0.8419 (5)	−0.0350 (2)	0.0746 (11)	
H2	0.1166	0.8883	0.0088	0.090*	
C3	0.10180 (15)	0.9206 (5)	−0.1017 (3)	0.0846 (13)	
H3	0.1125	1.0202	−0.1031	0.102*	
C4	0.08311 (15)	0.8515 (6)	−0.1688 (3)	0.0839 (13)	
H4	0.0813	0.9066	−0.2140	0.101*	
C5	0.06791 (14)	0.7066 (5)	−0.1681 (2)	0.0741 (11)	
H5	0.0563	0.6625	−0.2130	0.089*	
C6	0.06934 (11)	0.6201 (5)	−0.0995 (2)	0.0581 (9)	
C7	0.05310 (11)	0.4738 (4)	−0.09624 (19)	0.0551 (9)	
H7	0.0409	0.4271	−0.1401	0.066*	
C8	0.05470 (11)	0.3952 (4)	−0.0281 (2)	0.0571 (9)	
C9	0.03850 (13)	0.2431 (5)	−0.0214 (2)	0.0703 (11)	
H9	0.0268	0.1922	−0.0644	0.084*	
C10	0.03996 (14)	0.1718 (5)	0.0472 (3)	0.0789 (12)	
H10	0.0293	0.0724	0.0507	0.095*	
C11	0.05760 (14)	0.2473 (6)	0.1135 (2)	0.0798 (12)	
H11	0.0583	0.1969	0.1601	0.096*	
C12	0.07325 (13)	0.3910 (5)	0.1097 (2)	0.0689 (11)	
H12	0.0843	0.4392	0.1538	0.083*	
C13	0.07305 (11)	0.4701 (4)	0.0390 (2)	0.0578 (9)	
C14	0.19756 (12)	0.7990 (4)	0.28030 (18)	0.0503 (8)	
C15	0.16763 (13)	0.7107 (4)	0.3315 (2)	0.0628 (10)	
H15	0.1325	0.7115	0.3271	0.075*	
C16	0.18986 (15)	0.6257 (4)	0.3863 (2)	0.0716 (11)	
H16	0.1697	0.5686	0.4189	0.086*	
C17	0.24310 (16)	0.6224 (4)	0.3950 (2)	0.0708 (11)	
H17	0.2577	0.5645	0.4334	0.085*	
C18	0.27294 (14)	0.7039 (4)	0.3470 (2)	0.0638 (10)	
H18	0.3079	0.7009	0.3530	0.077*	
C19	0.25146 (12)	0.7941 (4)	0.28769 (18)	0.0514 (8)	
C20	0.28036 (11)	0.8773 (4)	0.23658 (18)	0.0524 (8)	
H20	0.3155	0.8749	0.2398	0.063*	
C21	0.25709 (12)	0.9640 (4)	0.18072 (18)	0.0504 (8)	
C22	0.28402 (14)	1.0520 (4)	0.1261 (2)	0.0639 (10)	
H22	0.3192	1.0524	0.1271	0.077*	
C23	0.25950 (17)	1.1351 (4)	0.0727 (2)	0.0737 (11)	
H23	0.2779	1.1914	0.0376	0.088*	
C24	0.20596 (17)	1.1363 (4)	0.0705 (2)	0.0734 (11)	
H24	0.1893	1.1932	0.0336	0.088*	
C25	0.17860 (13)	1.0551 (4)	0.1217 (2)	0.0632 (10)	
H25	0.1434	1.0584	0.1199	0.076*	
C26	0.20288 (12)	0.9648 (4)	0.17826 (18)	0.0514 (8)	
N1	0.08930 (10)	0.6160 (4)	0.03670 (17)	0.0675 (9)	
N2	0.17409 (9)	0.8829 (3)	0.22676 (15)	0.0528 (7)	
O1	0.0000	0.3361 (6)	−0.2500	0.1183 (16)	
O2	0.07381 (11)	0.8227 (5)	0.17081 (17)	0.1234 (14)	
H2A	0.0499	0.9004	0.2031	0.137 (18)*	
H2B	0.1071	0.8385	0.1869	0.137 (18)*	
H1	−0.0308 (14)	0.280 (6)	−0.248 (4)	0.205*	

### Atomic displacement parameters (Å^2^)

**Table:**

	*U*^11^	*U*^22^	*U*^33^	*U*^12^	*U*^13^	*U*^23^
C1	0.0437 (18)	0.066 (2)	0.064 (2)	0.0044 (17)	0.0016 (16)	−0.010 (2)
C2	0.065 (2)	0.075 (3)	0.084 (3)	−0.003 (2)	0.015 (2)	−0.021 (3)
C3	0.074 (3)	0.071 (3)	0.110 (3)	−0.002 (2)	0.029 (3)	−0.009 (3)
C4	0.077 (3)	0.095 (4)	0.080 (3)	0.006 (3)	0.017 (2)	0.008 (3)
C5	0.066 (2)	0.093 (3)	0.064 (2)	−0.002 (2)	0.0025 (18)	−0.005 (2)
C6	0.0389 (17)	0.073 (3)	0.063 (2)	0.0082 (17)	0.0009 (15)	−0.012 (2)
C7	0.0434 (18)	0.068 (2)	0.054 (2)	0.0046 (18)	−0.0042 (15)	−0.0172 (19)
C8	0.0383 (17)	0.062 (2)	0.071 (2)	0.0124 (17)	−0.0010 (16)	−0.016 (2)
C9	0.056 (2)	0.069 (3)	0.086 (3)	0.005 (2)	0.0002 (19)	−0.016 (2)
C10	0.058 (2)	0.067 (3)	0.112 (3)	0.011 (2)	0.010 (2)	0.003 (3)
C11	0.060 (2)	0.097 (4)	0.082 (3)	0.017 (2)	−0.002 (2)	0.010 (3)
C12	0.053 (2)	0.086 (3)	0.068 (3)	0.010 (2)	−0.0068 (18)	−0.003 (2)
C13	0.0365 (17)	0.071 (3)	0.066 (2)	0.0093 (17)	−0.0025 (15)	−0.011 (2)
C14	0.0470 (18)	0.047 (2)	0.0574 (19)	0.0011 (16)	0.0037 (16)	−0.0130 (17)
C15	0.054 (2)	0.063 (2)	0.072 (2)	−0.0028 (19)	0.0115 (18)	−0.010 (2)
C16	0.087 (3)	0.059 (3)	0.069 (2)	−0.006 (2)	0.013 (2)	−0.003 (2)
C17	0.087 (3)	0.063 (3)	0.063 (2)	0.002 (2)	−0.008 (2)	−0.003 (2)
C18	0.063 (2)	0.063 (2)	0.066 (2)	0.008 (2)	−0.0122 (19)	−0.009 (2)
C19	0.0486 (18)	0.049 (2)	0.0570 (19)	0.0012 (16)	−0.0049 (16)	−0.0135 (18)
C20	0.0380 (17)	0.054 (2)	0.065 (2)	−0.0018 (16)	−0.0014 (15)	−0.0157 (19)
C21	0.0512 (18)	0.0441 (19)	0.0560 (19)	−0.0004 (16)	0.0004 (16)	−0.0157 (17)
C22	0.064 (2)	0.061 (2)	0.066 (2)	−0.0080 (19)	0.0095 (19)	−0.014 (2)
C23	0.099 (3)	0.058 (3)	0.065 (2)	−0.011 (2)	0.011 (2)	−0.005 (2)
C24	0.095 (3)	0.059 (2)	0.066 (2)	−0.001 (2)	−0.015 (2)	−0.005 (2)
C25	0.060 (2)	0.057 (2)	0.073 (2)	0.0022 (19)	−0.0128 (19)	−0.011 (2)
C26	0.0475 (18)	0.047 (2)	0.060 (2)	0.0032 (16)	−0.0039 (16)	−0.0153 (18)
N1	0.0478 (16)	0.082 (2)	0.073 (2)	0.0054 (16)	−0.0043 (14)	−0.0156 (19)
N2	0.0406 (15)	0.0505 (17)	0.0672 (17)	0.0005 (13)	−0.0006 (13)	−0.0101 (15)
O1	0.077 (3)	0.118 (4)	0.160 (4)	0.000	0.014 (3)	0.000
O2	0.0619 (18)	0.204 (4)	0.104 (2)	−0.034 (2)	−0.0056 (17)	−0.013 (3)

### Geometric parameters (Å, °)

**Table:**

C1—C2	1.416 (5)	C14—N2	1.348 (4)
C1—C6	1.430 (5)	C15—H15	0.9300
C1—N1	1.365 (4)	C15—C16	1.354 (5)
C2—H2	0.9300	C16—H16	0.9300
C2—C3	1.362 (5)	C16—C17	1.414 (5)
C3—H3	0.9300	C17—H17	0.9300
C3—C4	1.414 (6)	C17—C18	1.359 (5)
C4—H4	0.9300	C18—H18	0.9300
C4—C5	1.350 (6)	C18—C19	1.429 (4)
C5—H5	0.9300	C19—C20	1.389 (4)
C5—C6	1.425 (5)	C20—H20	0.9300
C6—C7	1.371 (5)	C20—C21	1.388 (4)
C7—H7	0.9300	C21—C22	1.425 (5)
C7—C8	1.382 (5)	C21—C26	1.432 (4)
C8—C9	1.423 (5)	C22—H22	0.9300
C8—C13	1.434 (5)	C22—C23	1.356 (5)
C9—H9	0.9300	C23—H23	0.9300
C9—C10	1.358 (5)	C23—C24	1.414 (5)
C10—H10	0.9300	C24—H24	0.9300
C10—C11	1.420 (5)	C24—C25	1.359 (5)
C11—H11	0.9300	C25—H25	0.9300
C11—C12	1.345 (6)	C25—C26	1.426 (4)
C12—H12	0.9300	C26—N2	1.352 (4)
C12—C13	1.423 (5)	O1—H1	0.954 (11)
C13—N1	1.367 (5)	O2—H2A	1.093 (4)
C14—C15	1.430 (5)	O2—H2B	0.933 (3)
C14—C19	1.430 (4)		
			
C2—C1—C6	118.9 (4)	N2—C14—C19	122.5 (3)
N1—C1—C2	119.3 (3)	C14—C15—H15	119.7
N1—C1—C6	121.7 (3)	C16—C15—C14	120.7 (3)
C1—C2—H2	119.6	C16—C15—H15	119.7
C3—C2—C1	120.8 (4)	C15—C16—H16	119.4
C3—C2—H2	119.6	C15—C16—C17	121.2 (4)
C2—C3—H3	119.9	C17—C16—H16	119.4
C2—C3—C4	120.3 (4)	C16—C17—H17	120.0
C4—C3—H3	119.9	C18—C17—C16	119.9 (4)
C3—C4—H4	119.6	C18—C17—H17	120.0
C5—C4—C3	120.7 (4)	C17—C18—H18	119.4
C5—C4—H4	119.6	C17—C18—C19	121.2 (3)
C4—C5—H5	119.5	C19—C18—H18	119.4
C4—C5—C6	121.0 (4)	C18—C19—C14	118.5 (3)
C6—C5—H5	119.5	C20—C19—C14	118.2 (3)
C5—C6—C1	118.3 (4)	C20—C19—C18	123.3 (3)
C7—C6—C1	119.1 (3)	C19—C20—H20	119.8
C7—C6—C5	122.6 (3)	C21—C20—C19	120.4 (3)
C6—C7—H7	119.8	C21—C20—H20	119.8
C6—C7—C8	120.4 (3)	C20—C21—C22	123.8 (3)
C8—C7—H7	119.8	C20—C21—C26	117.8 (3)
C7—C8—C9	122.8 (3)	C22—C21—C26	118.4 (3)
C7—C8—C13	118.8 (3)	C21—C22—H22	119.2
C9—C8—C13	118.4 (4)	C23—C22—C21	121.5 (3)
C8—C9—H9	119.7	C23—C22—H22	119.2
C10—C9—C8	120.5 (4)	C22—C23—H23	120.0
C10—C9—H9	119.7	C22—C23—C24	120.0 (4)
C9—C10—H10	119.6	C24—C23—H23	120.0
C9—C10—C11	120.7 (4)	C23—C24—H24	119.7
C11—C10—H10	119.6	C25—C24—C23	120.6 (4)
C10—C11—H11	119.7	C25—C24—H24	119.7
C12—C11—C10	120.6 (4)	C24—C25—H25	119.4
C12—C11—H11	119.7	C24—C25—C26	121.2 (3)
C11—C12—H12	119.6	C26—C25—H25	119.4
C11—C12—C13	120.8 (4)	C25—C26—C21	118.2 (3)
C13—C12—H12	119.6	N2—C26—C21	122.7 (3)
C12—C13—C8	118.9 (4)	N2—C26—C25	119.1 (3)
N1—C13—C8	121.5 (3)	C13—N1—C1	118.5 (3)
N1—C13—C12	119.6 (3)	C14—N2—C26	118.4 (3)
C19—C14—C15	118.5 (3)	H2A—O2—H2B	107.1 (3)
N2—C14—C15	119.0 (3)		

### Hydrogen-bond geometry (Å, °)

**Table:**

Cg1 and Cg2 are the centroids of the C1/C6–C8/C13/N1 and C1–C6 rings, respectively.

**Table:**

*D*—H···*A*	*D*—H	H···*A*	*D*···*A*	*D*—H···*A*
O2—H2B···N2	0.933 (3)	1.942 (2)	2.873 (4)	175.2 (2)
C7—H7···O1	0.93	2.35	3.271 (4)	171.
O1—H1···O2^i^	0.95 (1)	1.98 (5)	2.777 (4)	139 (6)
C16—H16···Cg1^ii^	0.93	2.93	3.773 (5)	152
C18—H18···Cg2^iii^	0.93	2.93	3.848 (5)	168

Symmetry codes: (i) −*x*, −*y*+1, −*z*; (ii) *x*, −*y*+1, *z*+1/2; (iii) −*x*+1/2, −*y*+3/2, *z*+1/2.
